# The application of innovative ecosystems to build resilient communities in response to major public health events

**DOI:** 10.3389/fpubh.2024.1348718

**Published:** 2024-04-25

**Authors:** Juan Juan La, Man Li, Xiaolu Liu

**Affiliations:** ^1^School of Political Science and Public Administration, Xinjiang University, Ürümqi, China; ^2^Urban Construction College, Hebei Normal University of Science and Technology, Qinhuangdao, China

**Keywords:** innovative ecosystem, major public health events, community resilience, sequential game, Nash equilibrium

## Abstract

In recent years, major public health events have had a significant and far-reaching impact on communities. As a response, there has been an increasing interest in enhancing community resilience through innovative ecosystems that involve diverse stakeholders with varying needs and demands. This study investigates the application of innovative ecosystems to improve community resilience in the face of major public health events by utilizing a sequential game approach to balance the interests of government, community, and residents. Subsequently, a comprehensive questionnaire survey was conducted among key stakeholders to ascertain their objectives, requirements and concerns for the innovation ecosystem based on the analysis results of the game model. The reliability and effectiveness of the proposed research method were verified through the analysis and verification of the sequence game model and questionnaire survey results. Finally, according to our analysis results, we propose countermeasures for promoting innovative ecosystems to improve community resilience. The research results indicate that the successful implementation of innovative ecosystems requires consideration of the different needs of stakeholders such as government officials, community members, and residents. Combining these perspectives can effectively promote such systems while enhancing the community’s resilience to major public health events.

## 1 Introduction

In recent years, the global economy has been confronted with unprecedented challenges, notably exemplified by the profound impact of major public health crises such as the COVID-19 pandemic. These crises, characterized by their swift and unpredictable nature, have not only posed significant threats to public health but have also revealed the vulnerability of communities and the limitations of traditional intervention measures (1).

Historically, responses to health emergencies and pandemics have often relied on conventional strategies, including lockdowns and isolation measures (2). While these measures aim to mitigate the spread of diseases, they have raised concerns about their potential adverse effects on essential services, economic stability, and overall societal equilibrium (3, 4). These challenges have underscored the critical importance of enhancing community resilience to major public health events (5, 6).

The concept of community resilience, defined as a community’s ability to withstand, adapt to, and recover from disruptions, shocks, or crises, has been a growing focus of attention within academic and governmental circles. As major public health events continue to evolve, from historical pandemics to contemporary health emergencies, the need for a comprehensive and adaptive approach to fortify communities against such challenges has become increasingly apparent.

Concomitantly, the notion of innovative ecosystems has emerged as a strategic paradigm to address the complexities associated with enhancing community resilience (7). These ecosystems, characterized by dynamic networks of diverse stakeholders, including innovation entities, resources, innovation teams, government support, and infrastructure, offer a multifaceted and adaptive framework to strengthen communities in the face of unforeseen challenges (8, 9). The innovation ecosystem functions as an “energy exchange” hub that facilitates a dynamic “energy flow” (10). This conceptualization illustrates a intricate network where diverse entities collaboratively foster technological advancements and innovation, leveraging elements such as capital, talent, and technology as the vital lifeblood fueling inter-entity “energy exchanges” and resource pooling (11, 12). Scholarly contributions further analyze the structural and developmental trajectories of innovation ecosystems, emphasizing the pivotal roles of innovation agents, organizations, resources, and core enterprises in guiding the ecosystem toward growth and sustaining a synergy of mutual interests (13).

Considering the functional characteristics of the innovation ecosystem and its pivotal role in major public health events, this paper unveils the strategic stability of each game party and the influence of each element on strategy selection. Through a tripartite sequential game model involving the government, the community, and the residents, coupled with questionnaire surveys and statistical analyses, the study aims to provide valuable insights and countermeasure suggestions. This research holds substantial practical significance for promoting social and economic growth, improving market competitiveness, and advancing community economic progress.

The contributions of this study are as follows:

1. Employs the sequential game methodology to investigate the equilibrium in meeting the requirements of the government, the community, and the residents.
2. Investigates the utilization of innovative ecosystems for enhancing community resilience in the face of major public health events.
3. Uses a questionnaire-based approach to scrutinize and comprehend the objectives, requirements, and apprehensions of these stakeholders concerning the implementation of innovative ecosystem solutions.

This paper consists of six main parts, the first part is the introduction, the second part is the related works, the third part is the methodology, the fourth part is the results and analysis, the fifth part is the discussion and countermeasures, and the sixth part is the conclusion.

## 2 Related works

### 2.1 Research on community resilience assessment methods

This paper proposes a triadic conceptualization of community resilience as encompassing dynamic capabilities: coping capacity, intrinsic resilience, and transformative adaptation. The rationale for this triad is that communities facing a major public health event need not only the ability to respond to the current crisis (coping capacity), but also the intrinsic resilience to adapt to potential future crises and the ability to achieve long-term resilience through transformative adaptation. This triad takes into account the coping and adaptation needs of communities at different time scales, as well as the inherently dynamic and transformative nature of communities.

Some scholars, based on the connotation of community resilience, have identified assessment dimensions and constructed models for evaluating community resilience (14). They measured these attributes from four dimensions: technical, organizational, social, and economic, to assess community resilience. In addition to attributes, some scholars further divided community resilience into two aspects: resources and capabilities. The Community-Based Resilience Assessment (CoBRA) toolkit developed by the United Nations Development Programme’s Drylands Development Centre categorizes community resilience into community resources and community capabilities, assessing community resilience based on static community resources and dynamic community capabilities (15). Ma et al. (16) divided community resources and capabilities into four parts: community relationships, risk and vulnerability levels, emergency procedures, and community resource availability.

**Figure 1:**
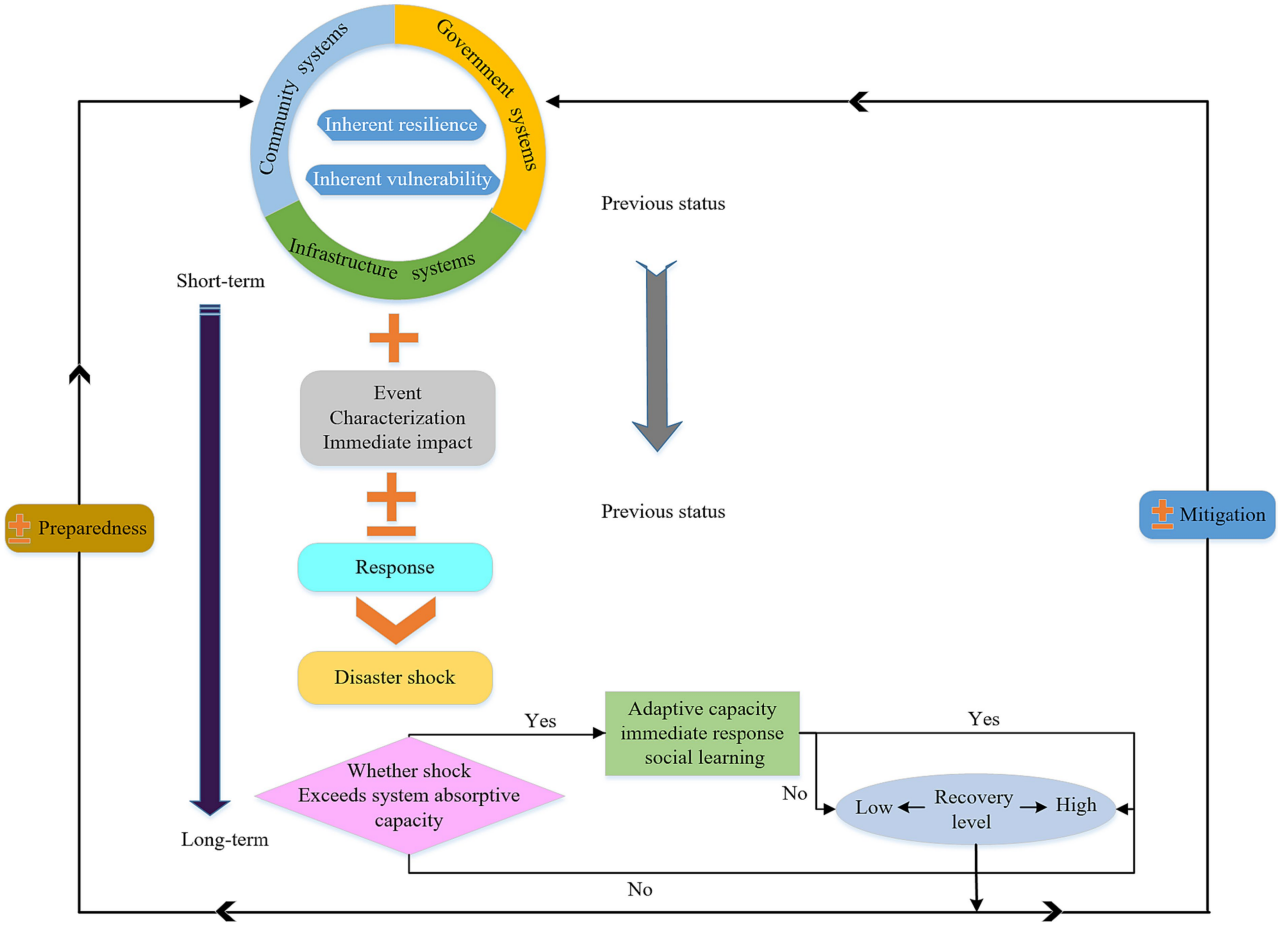
DROP model framework for public health events.

Lianxiao (17), based on the connotation of community resilience, proposed the principles of the Disaster Resilience Operational Principles (DROP) model for regional disaster resilience (as shown in Figure 1). The model explains the operational principles of community resilience before and after disasters. It suggests that the impact on a community is jointly determined by the community’s initial state, the direct impact of the disaster, and the immediate response the community can make. The model is applicable not only to sudden disasters like earthquakes but also to prolonged disasters such as droughts and public health events.

Some scholars posit that capital plays a pivotal role in community resilience, and it can be assessed and utilized to construct models by evaluating various forms of community capital. Wickes et al. (18) developed the community resilience model, categorizing community capital into economic and social capital. Economic capital primarily encompasses material capitals, such as community funds and supplies, while social capital focuses on social relationships. Paarlberg et al. (19) further detailed the categorization of community capital into social, economic, material, and human capital, distinguishing between material capitals and economic capital while also considering human capitals. Building upon this foundation, Koliou et al. (20) categorized community capital into human, infrastructure, natural capital, social, and economic capital, constructing a community resilience assessment model based on these five dimensions. Considering the context of communities amidst natural disasters, the evaluation extends to include the assessment of the natural environment and surrounding infrastructure of the community.

In addition, other scholars construct a community resilience assessment index system based on the constituent elements of the community from the perspective of the constituent elements of the community. While the primary focus is on internal community elements, it is crucial to acknowledge that communities are not isolated entities. Therefore, when summarizing community constituent elements, scholars usually also consider the community’s interactions with the outside world, especially the region in which the community is located. Camacho et al. (21) introduced the concept of the Community Baseline Resilience Index (BRIC) by Cutter, categorizing community constituent elements into infrastructure, ecosystems, institutions, economy, society, and community capital. Using these categories, they developed assessment indicators and quantitatively evaluated community resilience based on publicly available data for specific regions. Allen et al. (22) proposed the Resilience Matrix (RM) framework, employing a 4 × 4 matrix to evaluate community resilience across the disaster management stages of preparedness, absorption, recovery, and adaptation, focusing on infrastructure, communication, cognition, and social elements. Kammouh et al. (23) established the PEOPLES (People, Ecosystems, Organizations, Policies, Livelihoods, Economy, and Sociocultural Capital) framework, providing a detailed delineation of community constituent elements across seven dimensions.

As urban populations and building density increase, communities face escalating risks, posing more significant challenges to their crisis management capabilities. Following the outbreak of the COVID-19 pandemic in 2019, research into the systematic assessment of community resilience has placed a heightened emphasis on potential risk prevention and emergency governance, particularly in the context of major public health events. Wang et al. (24) employed interviews and grounded theory, explored the factors influencing community resilience during public health events, constructing a model based on dimensions such as volunteer participation, policy execution, autonomy, and consensus permeability. Dzigbede et al. (25) utilized an item response theory (IRT) model to categorize and delineate the concept of community resilience across four dimensions: capacity, attributes, structure, and processes, thereby establishing a theoretical model for community resilience assessment. Wang et al. (26), utilizing fuzzy analytic hierarchy process (FAHP) and fuzzy Technique for Order of Preference by Similarity to Ideal Solution (TOPSIS), developed a model for assessing community risk factors in the context of the COVID-19 pandemic. Using four communities as case studies, they quantified 12 evaluation indicators, including suspected infection, confirmed number, isolation of relevant personnel, enclosure and closure, and the establishment of unified entrances and exits, etc., resulting in safety risk values and relative rankings for the case study communities. Table 1 shows the community resilience assessment models under different construction bases.

**Table 1:** Summary of community resilience assessment models.

Author	Basis of model construction	Primary evaluation indicators
Koliou et al. (14)	Connotation of community resilience	Technical, organizational, social, and economic
Ma et al. (16)		Community relationships, risk and vulnerability levels, emergency Procedures, and community resource availability
Lianxiao et al. (17)		Static community resources and dynamic community capabilities
Wickes et al. (18)	Community capital	Economic capital and social capital
Paarlberg et al. (19)		Social capital, economic capital, material capital, and human capital
Koliou et al. (20)		Human capital, infrastructure capital, natural capital, social capital, and economic capital
Camacho et al. (21)	Constituent elements of the community	infrastructure, ecosystems, institutions, economy, society, and community capital
Allen et al. (22)		Infrastructure, communication, cognition, and social elements
Kammouh et al. (23)		People, ecosystems, organizations, policy, livelihood, economy, and sociocultural capital
Wang et al. (24)	Major public health events	Volunteer participation, policy execution, autonomy, and consensus permeability
Dzigbede et al. (25)		Capacity, attributes, structure, and processes
Wang et al. (26)		Suspected infection, confirmed number, isolation of relevant personnel, enclosure and closure, and the establishment of unified entrances and exits, etc.

### 2.2 Theory and research status of game theory

Game theory is a discipline that studies the behavior and decision-making of individuals in adversarial environments, which consists of three main elements: player, strategy, and payoff (27). It has been widely used in economics, psychology, mathematics, computer science and other fields (28). It can dissect strategic interactions and provide insights into optimal decision-making strategies and outcomes in situations where multiple actors influence each other. It explores situations where the outcome of an individual’s choice depends not only on their actions but also on the actions of others (29, 30). The fundamental concept revolves around modeling decision-making in scenarios where players, each pursuing their own objectives, must consider the potential reactions of others. The traditional game theory has gradually exposed its own problems in the research. It was not until the 1980s that scholars, addressing the assumptions’ flaws in traditional game theory, began seeking Nash equilibrium solutions under the “bounded rationality” assumption to overcome the limitations of traditional game theory research (31). And then dynamic games emerged as the mainstream research theory in game theory.

In Nash equilibrium, any unilateral change of strategy by any individual of the game will not bring better returns to itself, so the game will reach a certain stable state. The basic feature of sequential game is that the behavior of each player is in order. In most cases, the player who chooses later can observe the behavior of other players who choose earlier than him before he actually chooses. A salient attribute of a sequential game is the necessity for players to envision potential repercussions in forthcoming stages, utilizing this foresight to influence immediate choices. Moreover, ensuing players have the latitude to recalibrate their strategies predicated on the choices of their predecessors, thereby rendering the antecedent player’s actions a repository of fresh insights. Consequently, the quintessential challenge in a sequential game manifest in determining the optimum choice at every juncture, through the identification of the strategy sequence that yields the maximum benefit.

Game theory provides a methodology for emergency decision-making in the event of a critical incident outbreak to explore the interactive behavior among stakeholders. Table 2 illustrates the relevant studies by different scholars utilizing game theory under emergencies or disasters.

**Table 2:** Literature review of game theory modeling in emergencies and disasters.

Author	Year	Description
Georgalos (32)	2020	Constructed a centipede game model in order to analyze the cooperative behaviors among multiple subjects of emergency management under an unexpected disaster.
Kong et al. (33)	2022	Proposed a non-cooperative game model based on complete information.
Majumder et al. (34)	2023	Proposed an emergency resource scheduling model based on non-cooperative game, and used an improved ant colony optimization algorithm to find the solution.
Liu et al. (35)	2021	Used an evolutionary game to study the choices of the government and enterprises regarding the strategy of stockpiling the production capacity of emergency supplies, and proposed countermeasures to realize the cooperation between the two parties.
Chen et al. (36)	2023	Constructed a dynamic and finite sequential game by analyzing the relationship between decision makers and emergencies, and proposed the optimal rescue plan based on the emergency response information

### 2.3 Research on major public health events and their impact on communities, governments, and residents

A major public health event is defined as a sudden onset of a major infectious disease outbreak, mass unexplained disease, major food and occupational poisoning, or other event that seriously affects public health and causes or may cause serious damage to public health. The causes of these events are mainly pathogenic infections, food and drug safety, environmental pollution and natural disasters. Although they belong to the category of emergencies, major public health events also have their own special characteristics. In particular, unlike natural disasters, they are characterized by suddenness, hazard, urgency and variability.

**Figure 2:**
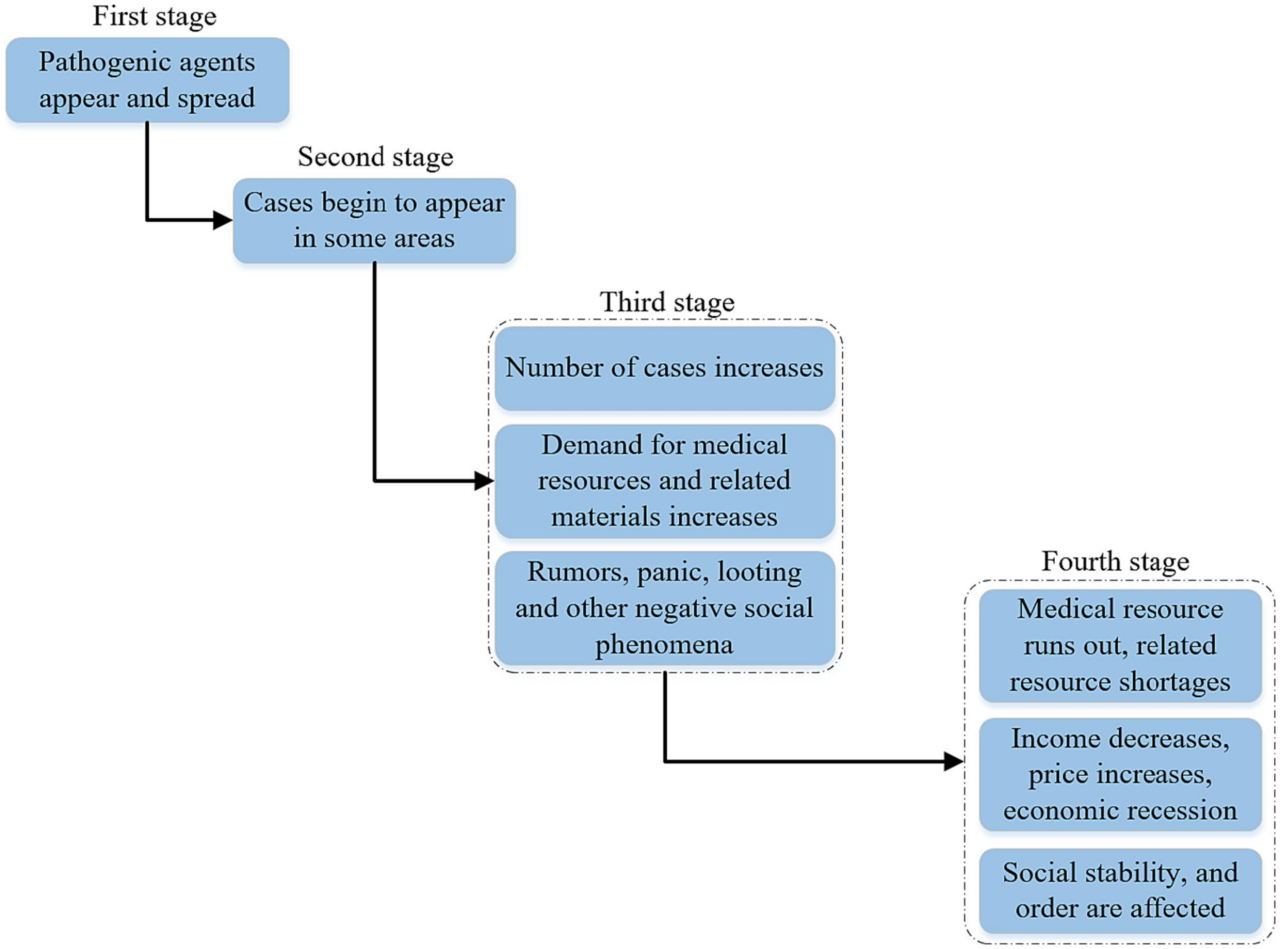
Stages of development of a major public health event.

Based on the risk warning level classified by the new crown epidemic data, some scholars have roughly divided the development process of major public health events into four phases based on the scope of impact and disaster losses, as shown in Figure 2.

In the first stage, the pathogenic agents are formed and spread through certain channels. In the second stage, the pathogenic agent begins to act on human bodies, cases begin to appear in some areas, and public health is threatened. In the third stage, the impact of the public health incident spreads further. In the fourth stage, the public health incident gradually worsens. And compared with the previous stage, public health events in this stage gradually get out of control. Resource inputs and social pressures continue to grow, and the impact of a major public health event not only spreads among populations and regions, but also spreads from the public health level to the overall social level, for example, the emergence of price increases, economic recession and social anxiety.

**Figure 3:**
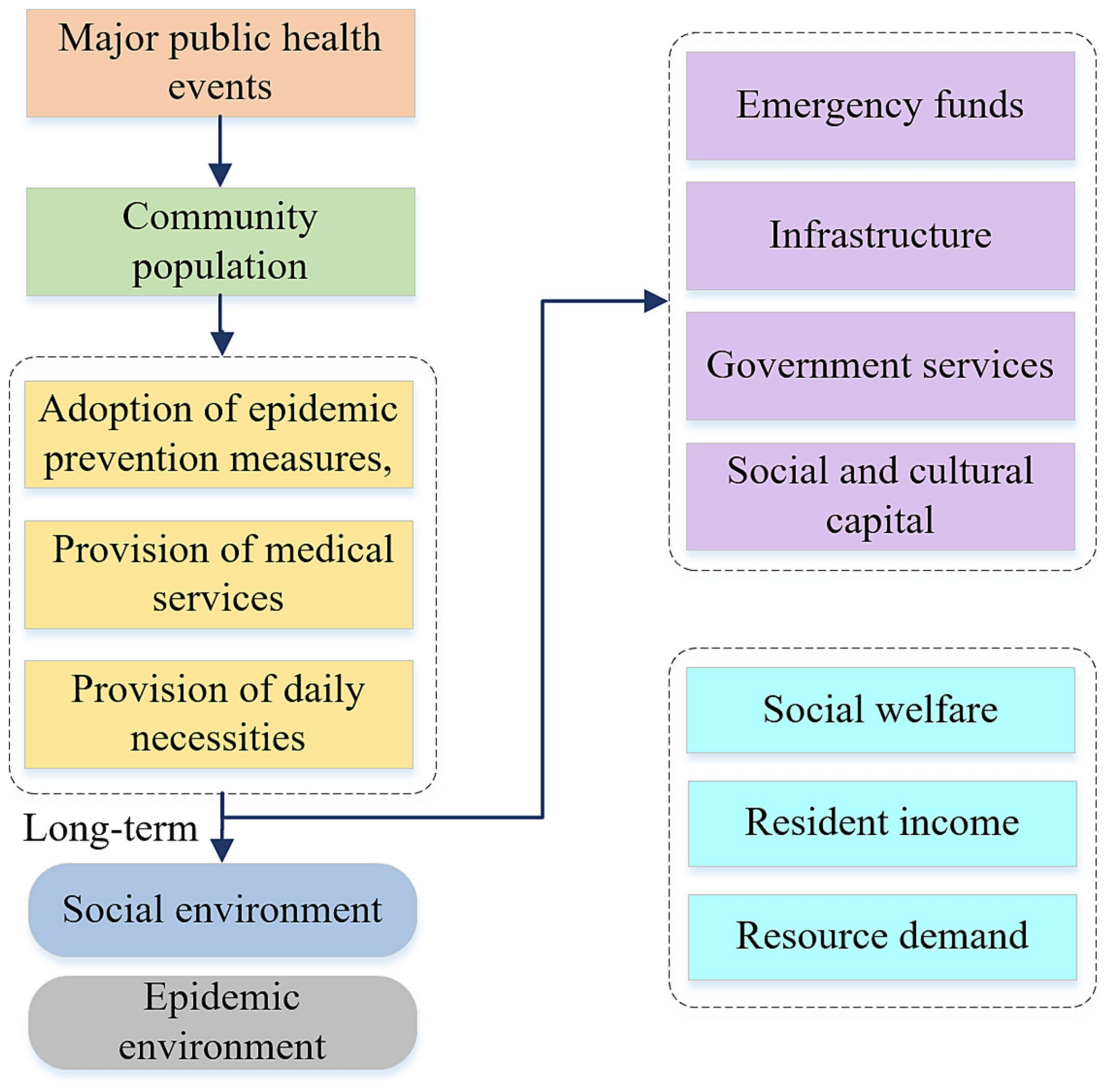
Impact mechanism of major public health events on communities and governments.

As the impact of a major public health event expands and deepens, not only does the availability of supplies for epidemic prevention and the living and medical needs of the population decline, but the employment and income of the population is also affected. When residents’ incomes are insufficient to maintain a basic life, the government and the community need to provide the necessary protection. Therefore, under a major public health event, the government is the main source of resource input. The interrelationship between them is shown in Figure 3.

## 3 Methodology

In this study, a tripartite sequential game model containing government agencies, community infrastructure and residents in hierarchical order is developed. Backward induction is then used to solve the sub-game fine Nash equilibrium problem of this tripartite sequential model. Based on the results of the analysis of the sequential game model, a set of questionnaires was designed with the aim of understanding the acceptance level of the tripartite measures aligned with the innovation ecosystem. Finally, the reliability of the questionnaire data was verified using Cronbach’s alpha coefficient, as well as KMO and Bartlett.

### 3.1 Game model building with interaction terms

As a kind of dynamic game, sequential game develops the actions of the participants into a tree-like graph due to the sequence of their actions (37). Different branches correspond to different decisions and benefit distribution. We enhance the original tripartite sequential game model by introducing interaction terms, specifically government-community interaction, government-residents interaction, and community-residents interaction.

Below is a simplified example of a three-way interaction model involving the government, community, and residents.

Original model:

Assuming the original tripartite sequential game model is represented as Formulas 1–6:


(1) $$U_{G}=f_{G}\;\left(\begin{array}{l} \mathrm{Government}’s\;\mathrm{decision,community}’s\;\mathrm{decision,}\;\\ \mathrm{residents}’\;\mathrm{decision} \end{array}\right)$$

(2) $$U_{C}=f_{C}\;\left(\begin{array}{l} \mathrm{Government}’s\;\mathrm{decision,community}’s\;\mathrm{decision,}\;\\ \mathrm{residents}’\;\mathrm{decision} \end{array}\right)$$

(3) $$U_{R}=f_{R}\;\left(\begin{array}{l} \mathrm{Government}’s\;\mathrm{decision,community}’s\;\mathrm{decision,}\;\\ \mathrm{residents}’\;\mathrm{decision} \end{array}\right)$$

Here, $U_{G}$ , $U_{C}\;$ , and $U_{R}$ represent the utilities of the government, community, and residents, respectively, while $f_{G}$ , $f_{C}$ , and $f_{R}$ denote their respective utility functions.

Building upon this foundation, we introduce interaction terms to capture the intricate relationships among government, community, and residents.


(4) $$U_{G}=f_{G}\;\left(\begin{array}{l} \mathrm{Government}’s\;\mathrm{decision,}\;\mathrm{community}’s\;\mathrm{decision,}\;\\ \mathrm{residents}’\;\mathrm{decision,}\;\mathrm{government}-\mathrm{community}\\ \mathrm{interaction,}\;\mathrm{government}-\mathrm{Residents interaction} \end{array}\right)$$

(5) $$U_{C}=f_{C}\;\left(\begin{array}{l} \mathrm{Government}’s\;\mathrm{decision,}\;\mathrm{community}’s\;\mathrm{decision,}\;\\ \mathrm{residents}’\;\mathrm{decision,}\;\mathrm{government}-\mathrm{community}\\ \mathrm{interaction,community}-\mathrm{residents interaction} \end{array}\right)$$

(6) $$U_{R}=f_{R}\;\left(\begin{array}{l} \mathrm{Government}’s\;\mathrm{decision,}\;\mathrm{community}’s\;\mathrm{decision,}\;\\ \mathrm{residents}’\;\mathrm{decision,}\;\mathrm{government}-\mathrm{residents}\\ \mathrm{interaction,}\;\mathrm{community}-\mathrm{residents interaction} \end{array}\right)$$

In these formulas, the interaction terms represent the impact of decisions made by one party on the utility of another. For instance, the government-community interaction term encapsulates how decisions by the government influence the community’s utility and vice versa.

The modified model now incorporates government-community interaction, government-residents interaction, and community-residents interaction. This enhancement aims to provide a more realistic representation of the interdependencies and mutual influences among the tripartite entities during major public health events.

**Figure 4:**
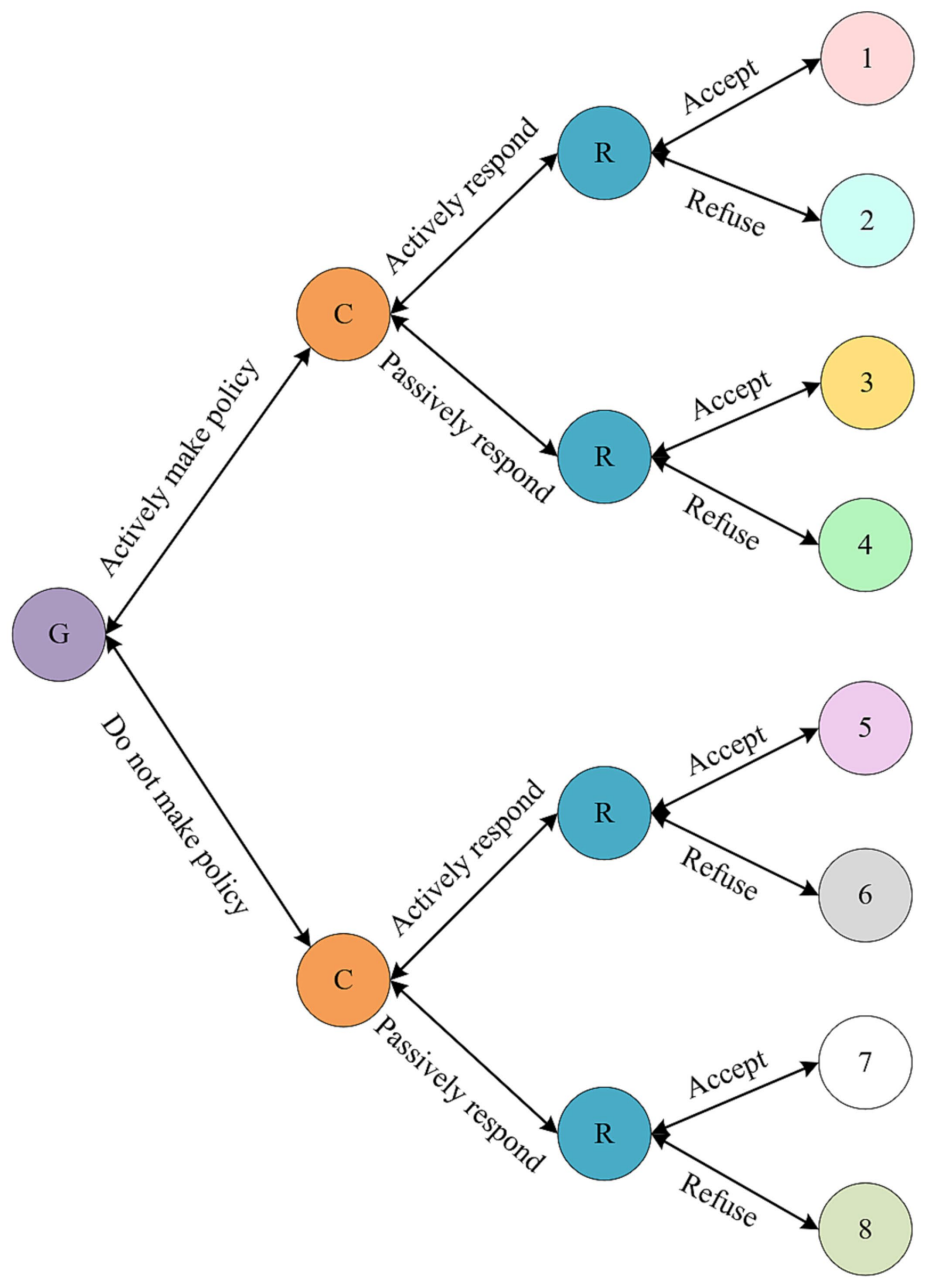
Tripartite sequential game model of government, community and residents.

The extended model is illustrated in Figure 4, showcasing the eight resulting game strategies formed by the choices of the government, community, and residents (numbered 1–8). As can be seen from Figure 4, when there are only two choices for the government, the community and the residents, eight game results will be formed, and the numbers 1–8, respectively, represent these eight game strategies (38).

### 3.2 Game strategy 1

The government makes policies actively, the community responds positively, and the residents accept the policies. In this case, a major public health event management system with the goal of building an innovative ecosystem will be formulated and implemented, and corresponding measures will be actively promoted in the community. Residents have a high degree of recognition of national policies and management and services provided by the community, and actively participate in and cooperate with them. In this case, the government won social reputation, balanced the contradiction between residents’ demand and social supply, and recorded the government benefit as R1. In order to promote the innovation ecosystem and enhance the resilience of the community in the face of major public health events, the government will allocate certain financial resources for corresponding construction, and the expenditure of the government is recorded as C1. Due to resource subsidence, the community will also receive corresponding benefits when implementing the policy, such as medical service benefits, supermarket service benefits, logistics distribution benefits, etc., and the sense of social responsibility and reputation will also increase accordingly, which is denoted as R2. In order to meet the national standards on the construction of resilient communities, create an innovative ecosystem, and improve residents’ satisfaction with community services when dealing with major public health events, communities need to establish corresponding implementation systems and allocate corresponding enterprises, personnel, facilities, etc., which requires a certain amount of capital expenditure, recorded as C2. Residents will be provided with relevant services needed to prevent and respond to major public health events, such as regular health tests, contact-free distribution services, unmanned sales services, intelligent logistics services, etc. Residents will greatly reduce the risk of collective transmission and reduce medical, transportation and living costs caused by major public health events. The above benefits are denoted as R3. However, if prices and medical expenses rise rapidly due to improper management of policies and communities after a major public health event, and then residents need to spend more to cooperate with the management of the government and community, residents will have to pay some additional expenses, recorded as E3.

### 3.3 Game strategy 2

The government makes policies actively, community hospitals respond positively, and residents do not accept the policies. At this time, in the context of the adoption of the innovative ecosystem, the corresponding measures of community protection and response to major public health events are actively promoted. However, due to the distrust of residents, information asymmetry of community behavior, and low satisfaction of residents on the services received, it is difficult to promote the relevant construction of the community, resulting in the loss of funds, enterprises and talents. Even the loss of residents, etc., at this time, the government’s policies have not realized the benefits, the income is 0; In order to promote the resilience of the community and the unresolved resource allocation problem, the government needs to pay a certain amount of financial expenditure, which is recorded as C1. Residents do not support and cooperate with the resilience construction of the community, resulting in the relevant benefits of the community is 0, and the cost of the community to respond to the national policy is recorded as C2. Because residents lack alternative ways to deal with major public health events, the benefit is zero. Meanwhile, the cost is zero because they do not cooperate with the relevant construction of the community.

### 3.4 Game strategy 3

The government makes policies actively, the community responds negatively, and the residents accept the policies. At this time, in the context of the risk of major public health events, the community expects that the community can actively respond to and implement national policies, so as to reduce the sense of panic and helplessness of residents in the face of major public health events, and improve the confidence and satisfaction of residents in dealing with major public health events. However, the community performs per functionally and slowly promotes the construction progress. The benefit to the government is zero. C1 is the expenditure required by the government to address the lack of resilience of communities to respond to major public health events. The community ignores the policy, resulting in the loss of social responsibility and reputation, and the decline in the revenue of various service industries in the community, etc., which is denoted as D2 and the benefit is 0. When residents enjoy the prevention and response system for major public health events supported by the innovative ecosystem, the benefit is R3. However, due to the lack of attention of the community, residents face the risk that it is difficult to obtain security services and humanistic care in the event of major public health events, and the loss of residents is recorded as E3.

### 3.5 Game strategy 4

The government makes policies actively, the community responds negatively, and the residents do not accept the policies. At this time, although the policy actively promotes the use of innovative ecosystem to prevent and respond to major public health events, the community and residents do not support and cooperate with the national policy, leading to the decline of the reputation of the government, the waste of resources and the failure to solve the problem. The benefit of the government is recorded as 0 and the expenditure as C1. The loss of social responsibility, reputation and residents caused by the community ignoring the policy is recorded as D2. Because no national policies were implemented, the community’s benefits were recorded as 0. Residents are not affected by the national policy, so the benefits and losses are 0.

### 3.6 Game strategy 5

The policy is not formulated, the community responds positively, and the residents accept the policy. At this point, the benefit of the government is 0 and the expenditure is also 0. The community is recognized by the residents by implementing an innovative ecosystem to prevent and respond to major public health events, and its sense of social responsibility, reputation and even occupancy rate will be improved. The benefit is denoted as R2. In order to improve community resilience, without financial support from the government, the community needs to pay more financial expenditure, which is recorded as F2. Although the residents did not enjoy the policy dividends of the government, they also benefited from the quality services in the face of major public health events, and even received more refined services and humanistic care, whose benefits were recorded as R3.

### 3.7 Game strategy 6

The government does not make a policy, the community responds positively, the residents do not accept the policy. At this time, the government ignored the waste of resources, price fluctuations, difficult to ensure the livelihood of residents and other problems, and the government benefit was 0. Due to the residents’ rejection, the occupancy rate, social responsibility and reputation of the community did not change significantly, and the benefit was recorded as 0. However, in order to attract residents, the community would have to pay more finance, which was recorded as C2. Residents do not accept the policy, the benefit and loss are 0.

### 3.8 Game strategy 7

The government does not formulate policies, the community responds negatively, and the residents accept the policies. At this time, the benefit of the government is 0, and the benefit of the community is 0. Residents cannot enjoy the benefits of the innovation ecosystem for the prevention and treatment of major public health events, and the loss is recorded as G.

### 3.9 Game strategy 8

The government does not formulate policies, the community responds negatively, and the residents do not accept the policies. At this time, government benefit, community benefit and resident benefit are all 0.

The eight game strategies proposed in the tripartite sequential game model are comprehensive, encompassing a spectrum of scenarios that may unfold during a significant public health crisis, where government, community, and residents interact. Each strategy contemplates diverse combinations of responses from the government, community, and residents, covering a wide range of situations that include both positive and negative reactions from all parties. This provides a thorough analysis of all possible outcomes in the context of government, community, and resident interactions during a major public health crisis.

According to the above analysis, a total of 8 game strategies are obtained, with income = benefit-expenditure for each party. See Table 3 for the summary of the results.

**Table 3:** Game results of government, community and residents.

Serial	Game strategy (G, C, R)	Benefit (G, C, R)
1	Actively make policy, actively respond, accept	R1-C1, R2-C2, R3-E3
2	Actively make policy, actively respond, refuse	-C1, -C2, 0
3	Actively make policy, passively respond, accept	-C1, -D2, R3-E3
4	Actively make policy, passively respond, refuse	-C1, -D2, 0
5	Do not make policy, actively respond, accept	0, R2-F2, R3
6	Do not make policy, actively respond, refuse	0, -C2, 0
7	Do not make policy, passively respond, accept	0, 0, −G
8	Do not make policy, passively respond, refuse	0, 0, 0

The meanings of variables and parameters involved in the model are shown in Table 4.

**Table 4:** Meanings of variables and parameters.

Variables	Meanings
U_G, U_C, U_R	Utilities of the government, community, and residents
f_G, f_C, f_R	Utility functions of the government, community, and residents
G	Government
C	Community
R	Residents
R1, R2, R3	Government benefits, community benefits, resident benefits
C1, C2	Government expenditures, community expenditures
D2	Loss of social responsibility, reputation, and residents due to community neglect of the policy
F2	Financial expenditure for community resilience
E3	Additional expenses for residents
-G	Loss for residents due to the absence of community policies
-C1, -C2, -D2	Losses for the government, community, due to various factors

### 3.10 Implementation of questionnaire survey

Based on the analysis results of the game model, this paper designs a set of questionnaires to investigate which measures in line with the innovation ecosystem can be accepted or not accepted by the government, the community and the community residents, and the main reasons for not accepting them. The questionnaire is divided into three categories, respectively for the government, community and residents three aspects. Each questionnaire contains 16 questions, among which 8 questions correspond to the advantages that the participants think the community adopts the innovative ecosystem to prevent and respond to major public health events under the eight game strategies, which can be selected from multiple options. 8 questions, respectively, correspond to the measures that can be taken to deal with major public health events in the innovative ecosystem under the eight game strategies. The options in the questionnaire are established based on the comprehensive scenarios derived from the eight game strategies proposed in the tripartite sequential game model. These strategies encompass a wide spectrum of situations that may arise during a significant public health crisis, involving interactions among the government, community, and residents. The influencing factors for the options in the questionnaire are derived from the advantages and measures corresponding to each game strategy. It is therefore broadly representative. The survey was carried out in Beijing, Hangzhou, Shanghai and Guangzhou. A total of 936 questionnaires were distributed. Questionnaires with an answer rate of less than 60%, i.e., 16 questions, in which the respondents omitted to answer more than 9 questions, were regarded as invalid questionnaires. After eliminating the invalid questionnaires, 895 valid questionnaires were recovered, with an effective rate of 95.6%.

The choice of Beijing, Shanghai, Guangzhou, and Hangzhou as study regions was based on several considerations. (1) Economic Significance: These cities are economic hubs in China, representing diverse industries and economic activities. Studying community resilience in such influential regions allows for broader applicability of findings. (2) Population Density: High population density and urbanization characterize these regions, making them pertinent for understanding the dynamics of community interactions during public health crises. (3) Innovation Ecosystem: These cities are known for fostering innovation ecosystems. Analyzing community resilience aligned with innovation ecosystems in these regions offers insights into the effectiveness of such strategies in advanced environments. However, the sample deliberately includes participants spanning various age groups, education levels, and health conditions, and it intentionally covers individuals from various socio-economic backgrounds, preventing coverage bias and ensuring a well-rounded representation. In summary, the selection of these regions is strategic and representative.

The demographic characteristics of the participants and information about the communities they governing or belonging to are shown in Table 5. It can be seen from Table 5 that more communities do not use or seldom use innovative ecosystem than those use it.

**Table 5:** Information about the participants and their communities.

Region	Category			Does the community you govern or belong to use innovative ecosystem		
	Governor	Community staff	Residents	Use	Have but seldom use	Do not have
Beijing	3	21	190	65	54	95
Hangzhou	4	16	192	32	38	142
Shanghai	6	28	205	75	61	103
Guangzhou	5	27	208	56	46	128

The basic profile of the participants in the questionnaire survey is shown in Table 6.

**Table 6:** Basic information on investigators.

Content	Variable	Frequency	Percentage
Gender	Male	450	50.3
	Female	445	49.7
Age	18 years old and below	22	2.5
	19–35 years old	362	40.4
	36-60 years old	385	43.0
	61–65 years old	76	8.5
	65 years old and above	50	5.6
Education level	Primary school and below	32	3.6
	Junior high school	128	14.3
	Senior high school and secondary specialties	216	24.1
	College or undergraduate	448	50.1
	Postgraduate and above	71	7.9
Physical health condition	Disease	9	1.0
	Chronic disease	48	5.4
	Sub-health	172	19.2
	Health	321	35.9
	Good health	345	38.5

As can be seen from Table 6, the questionnaire sample covers as many people as possible from 18 years old and below to over 65 years old in terms of age, in terms of education, the sample covers primary school, junior high school, senior high school and other educational levels, and in terms of physical health conditions, the sample includes people with diseases, sub-healthy people, as well as healthy people and other types of people. This shows that the sample has a balanced coverage in all aspects, without coverage error and selection bias, and is representative of the population as a whole.

## 4 Results and analysis

### 4.1 Game model and strategies analysis

Find the equilibrium point from the bottom up by backward induction. First, from the perspective of maximizing residents’ benefits, R3-E3 > 0, that is, when the cost of effective protection and response to major public health events is lower than the cost of direct impact of major public health events without protection, node 1 is selected between node 1 and node 2, and node 2 is selected otherwise. If R3-E3 > 0, node 3 is selected from node 3 and node sum; otherwise, node 4 is selected. R3 > 0, so choose node 5 between node 5 and node 6; −G < 0, so keep node 8 between node 7 and node 8.

Then from the perspective of benefit maximization of community hospitals, namely from node 1 and node 3, node 2 and node 4, node 5 and node 8 between the game selection. In node 1, the payoff for the community is R2-C2 and in node 3, the payoff is -D2. If R2-C2 > -D2, it indicates that the community’s social responsibility and reputation are greater than the construction cost and talent cost to attract residents, so node 1 is retained, and node 3 is retained vice versa. In node 2, the benefit of the community is -C2, and the benefit of node 4 is -D2. If -C2 > -D2, that is, the cost to be paid to improve the resilience of the community in response to major public health events is less than the loss of social responsibility and reputation caused by the community’s failure to carry out relevant construction, node 2 is selected; otherwise, node 4 is selected. Between node 5 and node 8, if R2-F2 is greater than 0, it indicates that the benefits gained by the community from actively improving the resilience of community protection and response to major public health events from the perspective of innovation ecosystem are greater than the benefits from the community’s negative response. Node 5 is selected, and node 8 is selected otherwise.

Finally, from the perspective of the government, Table 3 and mathematical equation show that from strategies 5 to 8, the government’s income is 0, and from strategies 2 to 4, the government’s income is -C1, and R1-C1 must be greater than -C1. From the perspective of the actual situation and the government’s formulation of strategies, the original intention of the government is to accelerate the construction of an innovative ecosystem to improve the resilience of communities to prevent and cope with major public health events, so that even if a major public health event occurs, residents can maintain a normal and orderly life, so the benefits must outweigh the costs. If the community chooses node 1, the government makes the same choice. If the community chooses 2, 3 or 4, then the government will have no income and will not develop policies to build the community.

It can be seen from the above that the Nash equilibrium points of the government-community-residents tripartite sequential game model are nodes 1, 5, and 8.

- If node 1 is selected, it indicates that the government actively formulates policies on community construction innovation ecosystem, the community responds positively, and the residents accept and cooperate actively. At this point, it shows that the government intends to promote the construction of the community innovation ecosystem, and provides financial support to people, money and other aspects. The community responds positively. If the benefit of both sides is greater than the expenditure, all three parties in the game can benefit.
- If node 5 is selected, it indicates that the government does not make policies, but the community and residents respond positively. At this time, the risk of major public health events is high, residents cannot get health care and other problems are prominent, the government does not do the corresponding top-level design, does not provide institutional support, the community will encounter multiple obstacles in promoting the innovation ecosystem, residents will not enjoy the corresponding policy dividends, in this case, both sides except the government can benefit.
- If node 8 is selected, it indicates that the government, community and residents do not promote the construction of community innovation ecosystem from top to bottom, and there are no institutions and no residents to respond and buy. At this time, there are still problems such as high risk of major public health events and lack of emergency measures. It is difficult to guarantee the health and order of life of the residents, and there are no beneficiaries.

From the perspective of Nash equilibrium, the survey results of game strategies 1, 5 and 8 are shown in Table 7. The main reasons why the government considers or is willing to develop policies to promote the innovation ecosystem to improve the resilience of communities in preventing and responding to major public health events include resource sharing, economic growth and innovation, while the main reasons why the government is reluctant to develop policies include excessive financial burden, high resource consumption and lack of mature technical system. The government has more recognition for the listed innovative ecosystem measures, such as the establishment of intelligent logistics system, the establishment of medical cloud platform, and the improvement of testing technology. In contrast, the main reasons that communities consider or are willing to implement the government’s plan to adopt innovative ecosystems to enhance community resilience in preventing and responding to major public health events include improved community well-being, improved community public safety, and improved community market competitiveness. However, the main reasons that communities are reluctant to implement the government’s policies on improving community resilience are too easy to implement, poor economic or material conditions of communities, and insufficient recognition of residents. Among the listed innovative ecosystem initiatives, the community more agreed with the establishment of intelligent logistics system, the establishment of medical cloud platform, and the centralized treatment of patients. For residents, the main reasons for their willingness to support and cooperate with governments and communities to build innovative ecosystems to prevent and respond to major public health events include benefits for personal health, benefits for livelihood security, benefits for continuity of work or study, The main reasons for residents’ reluctance to cooperate with the government or community to establish an innovative ecosystem include operational difficulties, rising living costs, and waste of time. Among the innovative ecosystem measures listed, residents were more likely to agree with the establishment of a smart logistics system, the provision of tax incentives and free vaccinations.

**Table 7:** Representative statistical results of game strategies.

Category	Willing to adopt innovative ecosystem			Unwilling to adopt innovative ecosystem		
	Option	Selected	Ratio	Option	Selected	Ratio
Government	A.Promoting resource sharing and improve social welfare structure	18	90.0%	A.Heavy financial burden	16	80.0%
	B.Promoting economy growth	20	100.0%	B.High resource consumption	15	75.0%
	C.Improving social innovation	19	95.0%	C.Lack of mature technical system	12	60.0%
	D.Promoting social transformation	16	80.0%	D.Gloomy market outlook	8	40.0%
	E.Promoting social sustainable development	16	80.0%	E.Undermine social equity	10	50.0%
	F.Promoting social stability	15	75.0%	F.Lack of mature regulation system	10	50.0%
Community	A.Enhance community competitiveness	73	79.3%	A.Low policy operability	62	67.4%
	B.Enhance the connection ability of community	70	76.1%	B.Insufficient resource condition of community	76	82.6%
	C.Promoting the innovation ability of community	88	95.7%	C.Low acceptance of residents	59	64.1%
	D.Promoting the happiness and satisfaction of residents	84	91.3%	D.Lack of technology and technicians	61	66.3%
	E.Promoting public safety of community	86	93.5%	E.Unacceptable for the social culture	43	46.7%
	F.Promoting the welfare of community	79	85.9%	F.Lack of reliable allocation model of interest	58	63.0%
Residents	A.Improving life quality	684	83.0%	A.High cost	614	74.5%
	B.Beneficial to personal health	785	95.3%	B.Not convenient	523	63.5%
	C.Beneficial to living security	744	90.3%	C.Too complex	436	52.9%
	D.Increase employment opportunity	662	80.3%	E.High energy consumption	542	65.8%
	E.Enrich leisure life	543	65.9%	F.Time waste	571	69.3%
	F.Raise house value	511	62.0%	G.Do not help much	488	59.2%

It should be divided into 6 sections and different sections correspond to different game strategies. The left vertical three sections correspond to game strategy 1, and the right uppermost section corresponds to game 5, and the right nethermost two sections correspond to game strategy 8.

### 4.2 Questionnaire survey verification

#### 4.2.1 Reliability test

Reliability refers to the stability, consistency and reliability of the questionnaire results. The higher the reliability, the more consistent the results of the questionnaire data are, the more reliable the questionnaire is, and the next step of the study can be carried out. In this paper, the Cronbach’s α coefficient (39) is used to test the reliability of the questionnaire data. If the Cronbach’s α coefficient is greater than 0.7, it indicates that the reliability of the questionnaire data is high and the quality is better; if the coefficient is greater than 0.6 and less than 0.7, it indicates that the data is valid; if the coefficient is less than 0.6, it indicates that the data is invalid and the questionnaire has poorer credibility. After testing, the Cronbach’s α coefficient of this questionnaire is 0.752, which indicates that the reliability of the questionnaire is good (as shown in Table 8).

**Table 8:** Cronbach’s α reliability statistics.

Number of items	Cronbach’s α coefficient
16	0.752

#### 4.2.2 Validity test

Validity refers to the truthfulness and accuracy of the study, also known as authenticity. The higher the validity, the more the results of the questionnaire are in line with the research objectives, the more reasonable the questionnaire data are, and the more the research objectives can be realized. In this paper, KMO (40) and Bartlett (41) methods are used for testing. Firstly, KMO test is conducted, if the KMO value is more than 0.8, it indicates that the data authenticity of the questionnaire is very good; if the questionnaire is between 0.8 and 0.7, it indicates that the validity of the questionnaire is good; if the value is more than 0.6 and less than 0.7, it indicates that the validity of the questionnaire is in a reasonable range; if the value is less than 0.6, it indicates that the design of the questionnaire has problems. Secondly, Bartlett’s test was conducted. In this test, the significance should be less than 0.05 to show that the validity is good. After the test, the KMO test of this questionnaire is 0.906, and the significance of Bartlett’s test is less than 0.05, which indicates that the questionnaire has a good validity, see Table 9.

**Table 9:** KMO and Bartlett test.

KMO value		0.906
Bartlett test	Myopic chi-square	2594.335
	Degree of freedom	235
	Significance	0

## 5 Discussion and countermeasures

### 5.1 Win-win is the goal of promoting innovation ecosystem to enhance community resilience

From the analysis results of sequential game and Nash equilibrium, it can be found that among the eight strategies of the sequential game, only when each party can benefit can the implementation of the innovation ecosystem be promoted in the community, so as to improve the resilience of the community to prevent and respond to major public health events. If the interests of the government, the community or the residents are damaged, the innovation ecosystem cannot be implemented smoothly. Even if it is implemented, it is difficult to achieve the expected goals and benefits. However, the government, communities and residents have different needs and considerations for the innovation ecosystem. The government pays more attention to the contribution of the innovation ecosystem to social stability and development. For example, in the face of major public health events, the adoption of the innovation ecosystem can more efficiently cooperate with the function of the medical system and maintain the stability of the social order. The community pays more attention to the improvement of brand benefits of the innovative ecosystem, thus attracting more residents and businesses to settle in. Residents pay more attention to the impact of the innovation ecosystem on their health, work, study, life and economy. Therefore, it is not easy to strike a balance between the interests of the government, communities and residents. However, from the analysis results of sequential game and Nash equilibrium, it can be found that win-win can be achieved, that is, the government actively formulates policies, communities implement policies, and residents accept policies. In this case, the government needs to start from the perspective of communities and residents, take protecting and improving the welfare of communities and residents as the basic consideration, maintain social stability and sustainable development as the basic goal, and fully combine the objective conditions and development needs to formulate corresponding policies. Only in this way can the policy be accepted by the community and residents. In the implementation of policies, the acceptance and recognition of innovative ecosystem measures by communities and residents should be fully taken into account. If financial and material conditions allow, measures with high recognition by communities and residents should be selected. In addition, in the process of promoting the innovation ecosystem, communities and residents should be involved as much as possible to provide jobs and quality services for residents, so as to improve their satisfaction and happiness.

### 5.2 Collaboration is the foundation for advancing innovation ecosystems and enhancing community resilience

From the analysis results of sequential game and Nash equilibrium, it can be found that in order to smoothly promote the innovation ecosystem into the community and improve the resilience of the community in the face of major public health events, the government, the community and residents need to support and cooperate with each other. If any party does not cooperate, the resistance to promote the innovation ecosystem will increase, so that it cannot be implemented. For an innovation ecosystem that aims to enhance community resilience, it is a comprehensive innovation net composed of multiple actors, including governments, enterprises, communities, residents and other organizations and individuals. Multi-party cooperation is one of the important conditions for the success of innovation ecosystem. On the basis of their own interests and goals, different subjects cooperate to form common interests and equal points to achieve a mutually beneficial relationship, jointly promote the development of the whole innovation ecosystem in cooperation, and provide a high-quality platform for resource sharing, industry construction, residents’ mutual assistance and open cooperation. The government needs to provide policy and financial support for the development of the innovation ecosystem, guide and coordinate the integration of all aspects of resources and promote the win-win situation of the industry. Communities need to provide relevant platforms for enterprises or facilities in the innovation ecosystem to guide and serve enterprises to implement national policies. Residents need to actively participate in the establishment and implementation of the innovation ecosystem, and timely feedback their feelings to the community and other departments to promote the improvement of the innovation ecosystem. In short, multi-stakeholder collaboration is one of the key elements needed to build an innovation ecosystem. Through multilateral cooperation, all parties can enhance their innovation capacity and level on the basis of mutual benefit and achieve common prosperity and development.

### 5.3 Innovation is the key to promoting innovation ecosystems and enhancing community resilience

From the analysis results of sequential game and Nash equilibrium, it can be found that governments, communities and residents all hope to promote community resilience through innovation. In the past few years, most countries or regions in the world have experienced at least one major public health event. Each experience has had some impact on local economic development and social stability. However, few regions have introduced innovative ecosystems to enhance their resilience to major public health events. The original health care and security systems are often backward or over-saturated. Therefore, it is necessary and urgent to adopt innovative ecosystems in order to enhance the resilience of communities in the face of major public health events. From the perspective of the government, the adoption of an innovation ecosystem, the promotion of innovative technologies and the implementation of innovative models can better respond to changes in social and economic development, enhance the vitality of social development and make better use of human resources, technologies and resources. From the perspective of the community, the adoption of innovation ecosystem can provide the community with a wide range of innovation resources, such as capital, technology, talent, market, etc., so as to expand the field of community productivity, improve the economic, social and environmental ecological benefits of the community, and provide a strong driving force for the development of the community. At the same time, a number of new innovative enterprises will emerge in the innovation process. These businesses will create more opportunities for the economy and jobs in the community. From the perspective of residents, the participation of residents in the implementation process of the innovation ecosystem can promote the openness and cooperation of the innovation ecosystem, make the innovation ecosystem more perfect and powerful in the process of continuous innovation, and finally enable the community to have stronger resistance and adaptability in the face of major public health events. In many ways, therefore, innovation is the key to advancing innovation ecosystems and enhancing community resilience.

### 5.4 Regulation is the guarantee for promoting innovation ecosystems to enhance community resilience

From the analysis results of sequential game and Nash equilibrium, it can be found that the lack of effective supervision is an important reason for the government, communities and residents to promote the innovation ecosystem. In the process of promoting the innovation ecosystem to the community, regulators, as the main body of public management, bear the important responsibilities of regulating market order, ensuring public safety and protecting social interests. For the government, setting up regulatory agencies can not only ensure the efficient and orderly implementation of the innovation ecosystem, but also maintain the credibility and image of the government in a good state. The community should also set up corresponding regulatory departments to supervise and manage the daily operation process of the innovation ecosystem, so as to detect problems early and deal with them in time, so as not to affect the use of residents. For residents, the operation of the innovation ecosystem is directly related to the quality of their daily life. Therefore, residents should also assume the role of supervision over the operation of the innovation ecosystem. When they find operational problems in the innovation ecosystem, they should timely report to the community or report to the government regulatory agency. In addition, the high level of professionalism, the ability to keep up with The Times and the accuracy of Far East behavior, as well as the ability to ensure compliance and safety during the construction and implementation of the innovation ecosystem to prevent possible risks and losses, all contribute to the resilience of communities in the face of major public health events. Furthermore, regulatory authorities have the advantage of fully implementing policies, providing policy guidance and stimulating market vitality to promote healthy and orderly development of communities and order in the event of major public health events.

## 6 Conclusion

This study utilizes a sequential game theoretical framework and a questionnaire methodology to thoroughly examine the potential barriers and effective strategies for cultivating an innovative ecosystem that enhances community resilience during significant public health events. The investigation delves into the perspectives of three key stakeholders: governmental authorities, community entities, and individual residents.

The study’s empirical evidence highlights three potential scenarios where interactions among key stakeholders in fostering an innovative ecosystem may reach a Nash equilibrium. Survey responses from a varied participant group, including policymakers, community workers, and citizens, reveal diverse motivations and decision-making patterns. The findings indicate a preference for different approaches to incorporating innovative ecosystem solutions, shaped by individual factors like regulatory ease, social status enhancement, and cost-effectiveness. Based on these insights, the study suggests recommendations centered on collaborative synergy, mutual benefit, innovation drive, and regulatory oversight. The analysis of questionnaire survey helps to provide valuable information on research objectives, solve research problems, and provide information for decision-making or further investigation. Findings, supported by robust statistical measures such as a Cronbach’s α coefficient of 0.752 and a KMO value of 0.906, underscore the reliability and validity of the survey instrument.

However, it is important to acknowledge the study’s limitations, with a primary concern being the potentially compromised precision in sequential game outcomes due to the constrained scope of assimilated data, offering a simplified representation of the intricate realities dictating actual participant choices. Future research endeavors should aim to capture a more comprehensive data tapestry for a more holistic analytical landscape. Additionally, the study underscores the significant role of regional disparities in influencing determinative processes and strategic formulations related to the utilization of innovative ecosystems in resilient community construction. Consequently, Future research should focus on improving methodology by using comprehensive datasets, diversifying sample geography, and considering regional nuances in fostering community resilience through innovative ecosystems. Moreover, future research will focus on enhancing model complexity to better understand government-community-resident interactions during public health crises, considering specific parameters, influencing factors, and refined modeling techniques.

## Data availability statement

The original contributions presented in the study are included in the article/supplementary material, further inquiries can be directed to the corresponding author.

## Author contributions

JL: Funding acquisition, Resources, Visualization, Writing – review & editing. ML: Data curation, Project administration, Funding acquisition, Writing – review & editing. XL: Writing – original draft, Writing – review & editing, Software, Validation.
